# Structural characterization of DynU16, a START/Bet v1-like protein involved in dynemicin biosynthesis

**DOI:** 10.1107/S2053230X21008943

**Published:** 2021-09-21

**Authors:** Sarah K. Alvarado, Mitchell D. Miller, Minakshi Bhardwaj, Jon S. Thorson, Steven G. Van Lanen, George N. Phillips

**Affiliations:** a Department of BioSciences, Rice University, 6100 Main Street, Houston, TX 77005, USA; b Department of Pharmaceutical Sciences, College of Pharmacy, University of Kentucky, Lexington, KY 40536, USA; c Center for Pharmaceutical Research and Innovation, College of Pharmacy, University of Kentucky, Lexington, KY 40536, USA; d Department of Chemistry, Rice University, 6100 Main Street, Houston, TX 77005, USA

**Keywords:** START/Bet v1 domain, dynemicin, helix-grip fold, DynU16

## Abstract

The 1.5 Å resolution crystal structure of DynU16, a protein identified in the dynemicin-biosynthetic gene cluster, is reported. The structure adopts a di-domain helix-grip fold with a uniquely positioned open cavity connecting the domains. The elongated dimensions of the cavity appear to be compatible with the geometry of a linear polyene, suggesting the involvement of DynU16 in the upstream steps of dynemicin biosynthesis.

## Abstract 2

The crystal structure of DynU16, a protein identified in the dynemicin-biosynthetic gene cluster of *Micromonospora chersina*, was determined using iodide phasing and reveals a di-domain helix-grip fold.

## 1. Introduction   

**Figure 1:**
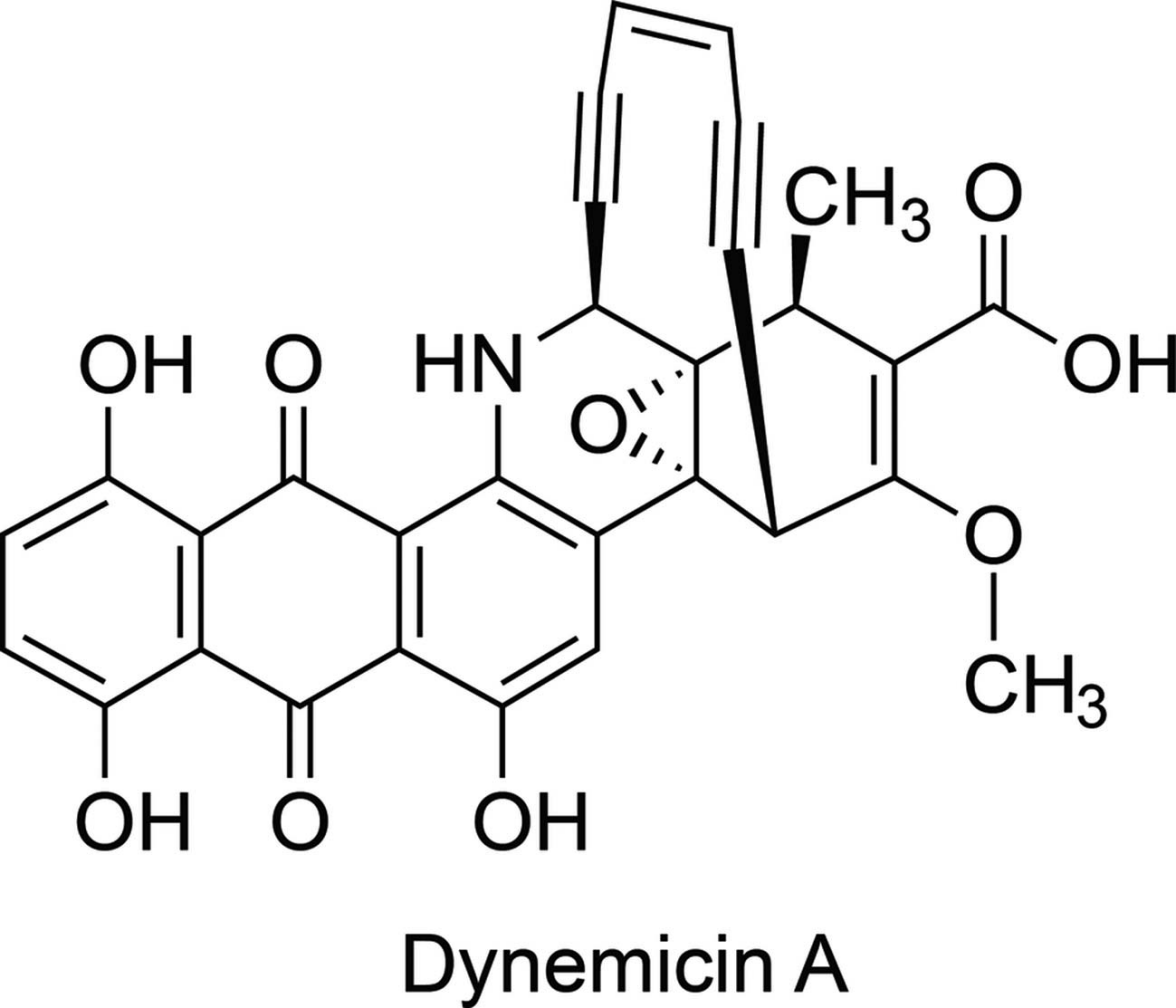
The chemical structure of dynemicin A, showing the enediyne ‘warhead’ above the anthraquinone-fused core. The figure was generated with *ChemDraw* (version 16; PerkinElmer Informatics).

Dynemicin A (DYN; Fig. 1 ▸) is a bacterial-derived metabolite possessing a ten-membered 1,5-diyn-3-ene core that achieves double-stranded DNA scission via radical-mediated hydrogen abstraction (Konishi *et al.*, 1990 ▸; Sluka *et al.*, 1987 ▸; Elbaum *et al.*, 1995 ▸). An anthraquinone (ANQ) moiety is appended to the core, with the quinone carbonyl O atoms serving as the activation sites for the reaction cascade, resulting in the formation of a phenyl biradical intermediate following core Bergman cyclization (Elbaum *et al.*, 1995 ▸; Nicolaou *et al.*, 1988 ▸; Sugiura *et al.*, 1990 ▸). The DYN-biosynthetic gene cluster was first reported in 2008, illuminating the conserved core-assembling elements encoded in other enediyne pathways (Gao & Thorson, 2008 ▸); however, many of the enzymes do not possess substantial homology to proteins in the Protein Data Bank (Berman *et al.*, 2003 ▸), raising more questions about DYN bioconstruction. The enediyne core is synthesized by a type I highly reducing iterative polyketide synthase (iPKS), yielding a linear conjugated polyene upon release from the iPKS by a thioesterase (Gao & Thorson, 2008 ▸; Ahlert *et al.*, 2002 ▸; Liew *et al.*, 2010 ▸). It was recently discovered that the iPKS also participates in the synthesis of the ANQ carbon skeleton, revealing the dual functionality encoded in the iPKS (Cohen & Townsend, 2018 ▸). The iPKS duality provides additional evidence supporting the notion that the tailoring enzymes govern the carbon-skeleton chemodiversity, as opposed to the iPKS and associated thioesterase (Horsman *et al.*, 2010 ▸). The downstream cyclases mediating enediyne-core cyclization have not been identified, and several potential candidates appear to be membrane-bound based on bioinformatic analysis, which impeded their functional and structural characterization. Fortunately, a subset of DYN enzymes share homology with enzymes encoded by aromatic polyketide pathways, which use cytoplasmic mono-domain and di-domain cyclase/aromatases to produce diverse decaketides.

Here, we report, at a resolution of 1.5 Å, the crystal structure of DynU16, a putative di-domain protein homolo­gous to StAR-related lipid-transfer (START/Bet v1-like) domain-containing proteins. Comparative bioinformatics to enediyne-biosynthetic gene clusters reveals homologues in the biosynthetic pathways of anthraquinone-fused enediynes. Moreover, DynU16 shares 30% sequence identity with CalU16, a calicheamicin mono-domain resistance protein; however, DynU16 possesses an additional domain, suggesting divergence in function from the self-sacrificing functionalities (Elshahawi *et al.*, 2014 ▸; Singh *et al.*, 2006 ▸; Biggins *et al.*, 2003 ▸). In search of a di-domain homologue, an aromatase BexL was identified from the BE-7585A aromatic polyketide pathway (Caldara-Festin *et al.*, 2015 ▸). In contrast to the type II PKS-associated di-domain cyclase/aromatases (CYC/AROs) BexL and StfQ, the DynU16 structure reveals an extended cavity bridging the domains. With only two reported di-domain structures from type II PKS systems, the DynU16 structure expands our limited knowledge.

## 2. Materials and methods   

### 2.1. Macromolecule cloning, expression and purification   

The DynU16 gene was codon-optimized for *Escherichia coli* expression and synthesized by Integrated DNA Technology (Coralville, Iowa, USA). The DynU16 gene was cloned into a pET-28a-derived expression plasmid via Polymerase Incomplete Primer Extension (PIPE) methods (Klock & Lesley, 2009 ▸). The PCR products were digested using the DpnI enzyme, mixed and annealed at 315 K. The annealed DynU16 and expression plasmid was transformed into NEB High-Efficiency *E. coli* cells (New England BioLabs, Ipswich, Massachusetts, USA) and plated onto kanamycin LB agar plates. Individual colonies were prepared and sequence-verified using Sanger sequencing (Genewiz, South Plainfield, New Jersey, USA). Positive clones were transformed into NEB *E. coli* BL21(DE3) expression cells.

A single colony was grown in LB medium supplemented with 50 µg ml^−1^ kanamycin overnight at 310 K. The cultured cells were inoculated into LB medium containing 50 µg ml^−1^ kanamycin and grown at 310 K until the OD_600_ reached ∼0.6. Protein synthesis was induced with 1.0 m*M* isopropyl β-d-1-thiogalactopyranoside (IPTG; GoldBio, St Louis, Missouri, USA) at 291 K for 18 h. The cells were harvested by centrifugation at 5000 rev min^−1^ at 277 K and stored at 193 K. For purification, the cells were thawed and resuspended in binding buffer consisting of 50 m*M* HEPES pH 7.5, 200 m*M* NaCl, 10 m*M* imidazole. Cell disruption was performed by sonication using a Qsonica sonicator programmed to 40% amplitude with iterative sonications. The lysed cells were separated by centrifugation at 18 000 rev min^−1^ for 45 min and the supernatant was incubated with 3 ml Nickel–NTA Superflow resin equilibrated with binding buffer (Qiagen, Hilden, Germany) on a rotating platform at 277 K. The protein-bound resin was washed with binding buffer supplemented with 30 m*M* imidazole and subsequently eluted with 200 m*M* imidazole. DynU16 was incubated with His-tagged TEV protease for 6 h at room temperature and further purified by reverse-phase nickel IMAC chromatography followed by size-exclusion chromatography on a HiLoad 16/600 column (GE Healthcare, Piscataway, New Jersey, USA). The protein concentration was determined from the absorbance at 280 nm using a calculated molar extinction coefficient of 75 970 *M* ^−1^ cm^−1^). Macromolecule-production information is summarized in Table 1 ▸.

### 2.2. Crystallization and derivatization   

DynU16 was concentrated to 15 mg ml^−1^ using an Amicon Ultra-15 centrifugal filter (Merck KGaA, Darmstadt, Germany) and subjected to high-throughput crystallization screening, which was performed using a Mosquito LCP robot (SPT Labtech) with the commercially available screens NeXtal JCSG Core Suites I–IV. Crystals were observed in well B13 of Core Suite III (Table 2 ▸, Supplementary Fig. S1). The crystals were cryoprotected with 20% glycerol and flash-cooled in liquid nitrogen. A heavy-atom derivative was produced by soaking crystals in 1 *M* potassium iodide for 1.5 min prior to cryoprotection with 10% glycerol and flash-cooling.

### 2.3. Data collection and processing   

The crystals were sent to the LS-CAT 21-ID-D beamline at the Advanced Photon Source (APS) for screening and data collection. A native data set was collected from a crystal that diffracted to a resolution of 1.5 Å and was indexed in space group *P*3_1_21. The data were integrated and scaled with *XDS* (Kabsch, 2010 ▸) within the *xia*2 (Winter, 2010 ▸) data-processing pipeline (Table 3 ▸). Data were collected from the heavy-atom-soaked DynU16 crystals at a wavelength of 2.07 Å to enhance the anomalous signal from iodide; the crystals diffracted to 2.3 Å resolution.

### 2.4. Structure solution and refinement   

DynU16 phases were determined by single isomorphous replacement with anomalous scattering (SIRAS) using *SHELXC*/*D*/*E* (Sheldrick, 2010 ▸) for anomalous signal assessment and phase calculations within the *HKL*2*MAP* graphical interface (Pape & Schneider, 2004 ▸). *ARP*/*wARP* was used for initial modeling building (Langer *et al.*, 2008 ▸). *Coot* (Emsley *et al.*, 2010 ▸) and *phenix.refine* (Liebschner *et al.*, 2019 ▸) were subsequently used for rebuilding and structure refinement (Table 4 ▸). The structure was visualized using a collaborative 3D graphics system (Yennamalli *et al.*, 2014 ▸). The *super* algorithm in *PyMOL* (version 2.4.1; Schrödinger) was used for r.m.s.d. calculations. The structural biology software applications used in this project were compiled and configured by SBGrid (Morin *et al.*, 2013 ▸). Coordinates and structure factors were deposited in the worldwide Protein Data Bank (Berman *et al.*, 2003 ▸) as PDB entry 6v04.

## 3. Results and discussion   

### 3.1. Overall structure   

**Figure 2:**
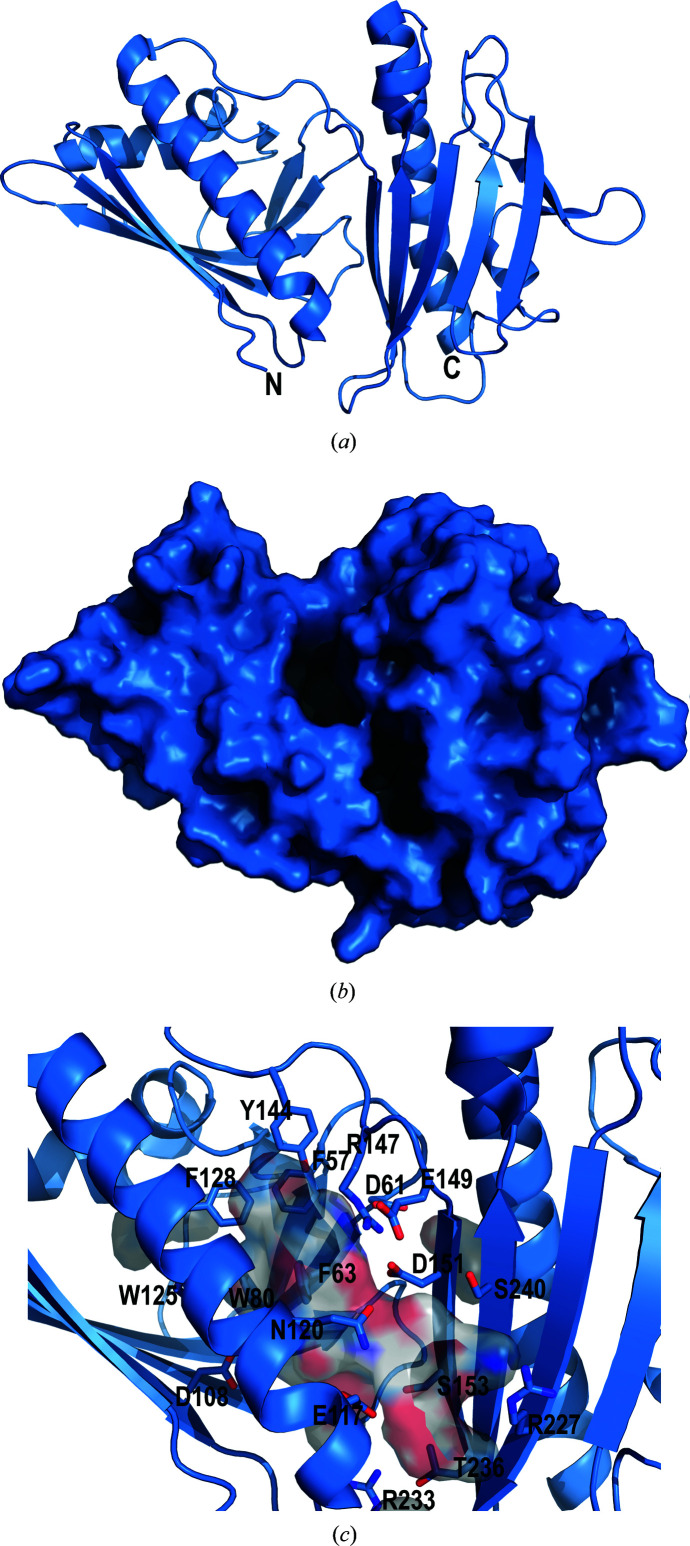
(*a*) DynU16 (PDB entry 6v04) adopts a helix-grip fold with a di-domain architecture. (*b*) The surface view shows the cavity formed between the N-terminal domain (left) and the C-terminal domain (right). (*c*) The elongated cavity is composed of aromatic residues localized towards the N-terminal domain and polar residues towards the C-terminal domain. The aromatic residues are positioned to stabilize the hydrophobic polyene substrate. The side chains lining the cavity are shown as sticks in cartoon representation, with C atoms shown in sky blue, N atoms in dark blue and O atoms in red. The surface of the cavity is shown to illustrate its size between the domains and the two openings. The figure was generated with *PyMOL* (version 2.4.1; Schrödinger).

The DynU16 structure was determined at a nominal resolution of 1.5 Å using SIRAS, revealing a di-domain helix-grip fold (Fig. 2 ▸ *a*, Table 4 ▸). The final model included 269 protein residues, 282 water molecules and five ions. Residues 0 (left from the tag after cleavage) to 8 at the N-terminus and 278–283 at the C-terminus were disordered and were not modeled. The N-terminal domain comprises an antiparallel β-sheet, two short α-helices and one extended α-helix. The N-terminal (residues 1–139) and C-terminal (residues 150–283) domains are connected by a flexible ten-residue linker (140–149). The sequence and structure similarity between the N- and C-terminal domains suggests that the second domain was acquired through a gene-duplication event. A structure-based backbone-atom alignment of 313 atoms in the N- and C-terminal domains was calculated, giving a root-mean-square atomic position deviation of 2.0 Å with 15% sequence identity over 107 aligned residues (Supplementary Fig. S2). Assessment of the C-terminal domain suggests divergence from the canonical START/Bet v1 fold observed in the N-terminal domain. The C-terminal domain possesses eight instead of six antiparallel β-strands and has adopted additional loops.

### 3.2. Putative cavity   

The cavity bridges the N- and C-terminal domains, with approximate dimensions of 22 × 12 × 8 Å (Figs. 2 ▸ *b* and 2 ▸ *c*). One end of the pocket comprises the polar residues Ser153, Ser240, Arg147, Arg227, Arg233, Glu117, Glu149, Asp61, Asp108, Asp151 and Gln120, along with Leu121. The back of the pocket has two clustered cysteines, Cys89 and Cys110, that are not engaged in a disulfide bond and could be involved in substrate stabilization. The opposing region of the cavity is composed of aromatic residues, including Phe57, Phe63, Phe82, Phe128, Trp80, Trp125 and Tyr144. The abundance of aromatic residues localized in one region of the cavity suggests a possible role in carbocation intermediate stabilization if cyclization is catalyzed in the cavity (Dougherty, 1996 ▸). The cavity-forming residues frequently reside in loops or β-strands, potentially increasing cavity flexibility and enabling contraction and expansion to accommodate substantial substrate rearrangements. The shape and size of the cavity appears to support the binding of a linear or perhaps a cyclic polyene (Fig. 2 ▸ *c*). The cavity openings (Fig. 2 ▸ *b*) could accommodate either a free polyene or one tethered to an acyl carrier protein (ACP) domain of the iPKS. However, it has not yet been established whether the enediyne core is synthesized attached to the ACP of the iPKS (Cohen & Townsend, 2018 ▸) or whether the intermediates remain free after they have been cleaved from the iPKS by the thioesterase (Annaval *et al.*, 2017 ▸).

### 3.3. Sequence and structure comparison   

**Figure 3:**
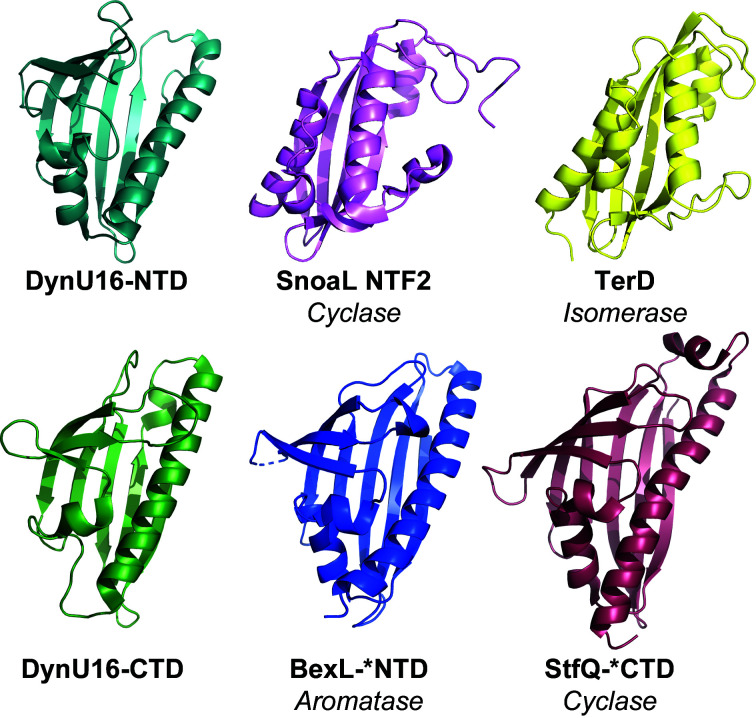
Structural comparison of the N-terminal domain (NTD) and C-terminal domain (CTD) of DynU16 with similar folds encompassing diverse functionalities including cyclization, aromatization and isomerization. The DynU16 helix-grip fold shares distant structural homology to the START di-domain polyketide-biosynthesis cyclase StfQ (PDB entry 4xrt; Caldara-Festin *et al.*, 2015 ▸) and aromatase BexL (PDB entry 4xrw; Caldara-Festin *et al.*, 2015 ▸). Additionally, the helix-grip fold resembles enzymes in the NTF2-domain family, including SnoaL, a mono-domain cyclase (PDB entry 1sjw; Sultana *et al.*, 2004 ▸), and TsrD, a mono-domain isomerase (PDB entry 5x7l; Y. Song, Z. Lin, W. Deng & W. Liu, unpublished work). The catalytic domains selected for illustration are marked with an asterisk. The figure was generated with *PyMOL* (version 2.4.1; Schrödinger).

Prior to the structural determination of DynU16, a limited number of structures of di-domain START/Bet v1 enzymes had been reported. BexL and StfQ were identified in type II PKS systems and operate on nonreduced and partially reduced decaketide substrates, respectively. BexL possesses aromatase activity localized to the N-terminal domain and a pseudocatalytic C-terminal domain. StfQ possesses a pseudo-catalytic N-terminal domain and cyclase activity localized to the C-terminal domain. In the cases of BexL and StfQ, the pseudo-catalytic domains are hypothesized to provide both solubility and structural support (Caldara-Festin *et al.*, 2015 ▸). Due to the limited information on the START-domain family, folds reminiscent of the START helix-grip fold were explored. The nuclear transport factor 2 (NTF2)-like cyclase α+β structure adopted by SnoaL and the isomerase TsrD provide additional evidence for the speculation that DynU16 harbors cyclase or dehydratase functionality (Fig. 3 ▸; Caldara-Festin *et al.*, 2015 ▸; Sultana *et al.*, 2004 ▸). The biosynthesis of the nine-membered enediyne antibiotic C-1027 requires SgcJ, which is a monodomain NTF2-like superfamily member (Huang *et al.*, 2016 ▸).

The NCBI *BLAST* platform (Altschul *et al.*, 1997 ▸) was used to explore the sequence space of related enzymes (Supplementary Fig. S3). The search identified 20 uncharacterized di-domain homologues with sequence identity equal to or exceeding 30% and 90% coverage, with *E* values of less than 2 × 10^−40^. Using *ESPript* (Robert & Gouet, 2014 ▸), an extended motif in the N-terminal domain, R*xx*F*x*DGDFF, was identified amongst related family members. Structural mapping of the conserved motif locates it to the connection between β-strands 3 and 4 in proximity to the cavity. This region of the cavity aligns with the polyketide-binding location in the bifunctional mono-domain ARO/CYC enzymes TcmN and WhiE (Lee *et al.*, 2012 ▸). Assessment of the interactions of the motif with nearby residues identifies a number of salt links between Arg147 and Asp151 and between Arg54 and Asp56 and van der Waals interactions between Phe63 and Trp80, suggesting that this motif may be involved in stabilizing the enzyme during catalysis. Other cyclase systems have been examined for their associated energetics, revealing highly exothermic reactions that could destabilize the enzyme without the proper network of stabilizing forces (Thoma *et al.*, 2004 ▸). Additionally, conserved residues were identified in the C-terminal domain, identifying a region that potentially mediates protein–protein interactions within the biosynthetic network. While an actual enzymatic assay is needed to confirm the speculated function of DynU16, we conclude that it is likely that this protein participates in the biosynthesis of dynemicin as a cyclase or perhaps at least as a dehydratase of an extended or cyclic ANQ precursor.

## Supplementary Material

PDB reference: DynU16, 6v04

Supplementary Figures. DOI: 10.1107/S2053230X21008943/rf5034sup1.pdf

## Figures and Tables

**Table 1:** Macromolecule-production information

Source organism	*Micromonospora chersina*
DNA source	*E. coli* codon-optimized gene synthesis, Integrated DNA Technology (Coralville, Iowa, USA)
I-PIPE forward primer	catgaaaacctgtacttccaatccATGCCAGATGGGTATTTGAAGCTT
I-PIPE reverse primer	ggatccgttatccacttccaatatt**tta**CGGCAATTCTTGGCGGGTACG
V-PIPE forward primer	CGTACCCGCCAAGAATTGCCGTAAAATATTGGAAGTGGATAACGGATCC
V-PIPE reverse primer	AAGCTTCAAATACCCATCTGGCATGGATTGGAAGTACAGGTTTTCATG
Expression vector†	pET His6 TEV LIC cloning vector (1B)
Expression host	*E. coli* BL21(DE3)
Complete amino-acid sequence of the construct produced‡	mgsshhhhhhenlyfq/sMPDGYLKLPDDWVRVMVSVPAPVDEVWEAVTDPRRVAQWFGHLSAPMTTGASTRVDFGDGDFFDIEVDHVEPRDRLLFRWSFLGVGPECQVGWTLTGGAEATTLTVDDSCPGRPGSEVAQLKAGWLDFVGRLARYLETGKPSRYDWRQEIDGSVVLPNGSWHPLREETVVDWLPIATNGAGPGWFFVVDEEGPRRFTLRDWQLDRERALTFAVEIPGARTVTACQVRTEPGERGRTLSVSHQGWHRLGLSDLQERTLRHRFAATWTAALSLAEECARTRQELP

† The pET His6 TEV LIC cloning vector (1B) was a gift from Scott Gradia (Addgene plasmid #29653).

‡ The expression and purification tag is shown in lower case, with the TEV recognition site underlined and the cleavage site indicated with a slash.

**Table 2:** Crystallization

Method	Vapor diffusion, sitting drop
Plate type	MRC 2 well UVP (SwissSci)
Temperature (K)	293
Protein concentration (mg ml^−1^)	15
Buffer composition of protein solution	20 m*M* HEPES pH 7.5, 200 m*M* NaCl
Composition of reservoir solution	100 m*M* Tris–HCl pH 8.5, 200 m*M* MgCl_2_, 20%(*w*/*v*) PEG 8000
Volume and ratio of drop	1 µl:1 µl
Volume of reservoir (µl)	200

**Table 3:** Data collection and processing Values in parentheses are for the outer shell.

	Native	KI soak
Diffraction source	APS beamline 21-ID-D	APS beamline 21-ID-D
Wavelength (Å)	1.00	2.07
Temperature (K)	100	100
Detector	Dectris EIGER X 9M pixel	Dectris EIGER X 9M pixel
Crystal-to-detector distance (mm)	180.1	100.0
Rotation range per image (°)	0.2	0.2
Total rotation range (°)	348	321
Space group	*P*3_1_21	*P*3_1_21
*a*, *b*, *c* (Å)	73.17, 73.17, 123.94	72.46, 72.46, 125.11
Resolution range (Å)	44.3–1.50 (1.54–1.50)	56.1–2.28 (2.42–2.28)
Total No. of reflections	1009253 (20881)	269410 (27463)
No. of unique reflections	58838 (2572)	17898 (2772)
Completeness (%)	95.4 (57.0)	99.4 (96.9)
Multiplicity	17.2 (8.1)	15.0 (9.9)
Mosaicity (°)	0.07	0.10
〈*I*/σ(*I*)〉	31.2 (1.6)†	12.1 (1.6)
*R*_meas_	0.044 (1.069)	0.149 (0.903)
CC_1/2_	1.00 (0.78)	1.00 (0.87)
Overall *B* factor from Wilson plot (Å^2^)	34	49

† 〈*I*/σ(*I*)〉 falls below 2.0 at 1.55 Å resolution.

**Table 4:** Structure refinement Values in parentheses are for the outer shell.

PDB entry	6v04
Resolution range (Å)	34.6–1.50 (1.54–1.50)
Completeness (%)	95.3
σ Cutoff	*F* > 0σ(*F*)
No. of reflections, working set	56687 (2134)
No. of reflections, test set	2087 (79)
Final *R*_cryst_	0.162 (0.277)
Final *R*_free_	0.188 (0.328)
No. of non-H atoms	
Protein	2129
Ions (Na/Mg/Cl)	1/1/3
Water	282
Total	2416
R.m.s. deviations	
Bonds (Å)	0.005
Angles (°)	0.8
Average *B* factors (Å^2^)	
Protein	35
Ions (Na/Mg/Cl)	26/23/33
Water	44
All-atom clashscore	1.6
Ramachandran plot	
Most favored (%)	96.3
Allowed (%)	100
