# Supplementary material for: Structural characterization of DynU16, a START/Bet v1-like protein involved in dynemicin biosynthesis
Source: Acta Crystallogr F Struct Biol Commun. 2021 Sep 21;77(Pt 10):328–33. doi: 10.1107/S2053230X21008943 (PMC8488855; doi:10.1107/S2053230X21008943)
Supplement: Supplementary file 1 [file f-77-00328-sup1.pdf]

Acta Cryst F
STRUCTURAL BIOLOGY
COMMUNICATIONS

Volume 77 (2021)

Supporting information for article:

Structural characterization of DynU16, a START/Bet v1-like protein involved in dynemicin biosynthesis

Sarah K. Alvarado, Mitchell D. Miller, Minakshi Bhardwaj, Jon S. Thorson, Steven G. Van Lanen and George N. Philips

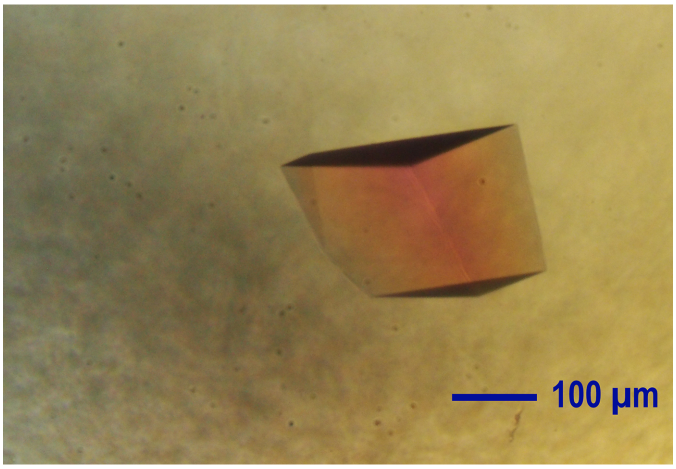

Figure S1 Crystal of DynU16 imaged with an Olympus BHTP polarizing microscope equipped with an Olympus OM-D E-M5 digital camera.

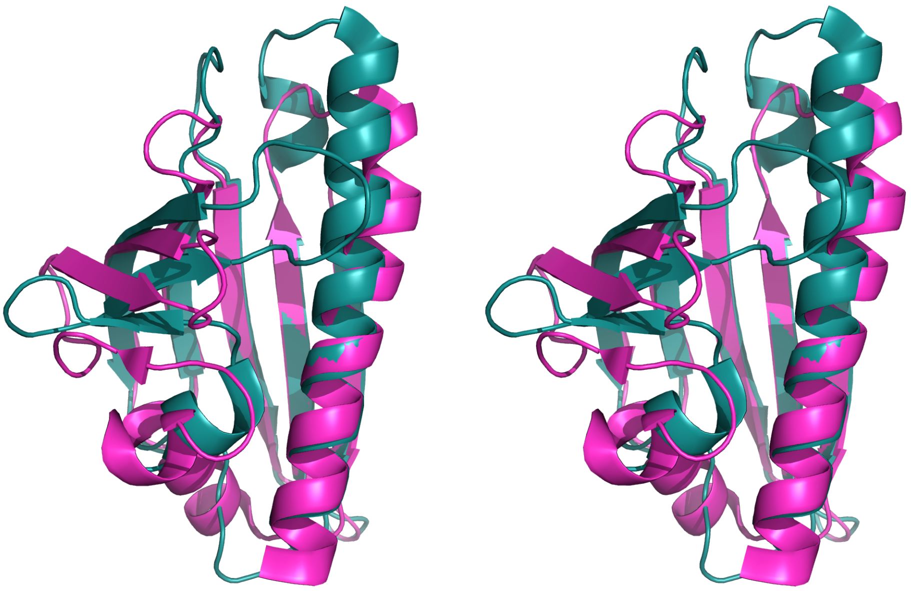

```
            β1            α1        α2      β2          β3
Nterm                                               TT
      9          20          30          40          50          60
Nterm PDDWVRVMVSVPAPVDEVWEAVTDPRRVAQWFGHLS....APMTTGASTRVDFGDGD...
Cterm IDGSVVLPNGSWHPL........REETVVDWLPIATNGAGP.......GWFFVVDEEGPR
    150          160                170          180          190
Cterm
          β8                         α4     β9          β10

            β4           β5                  β6          β7
Nterm                                                          TT
                  70          80          90         100         110
Nterm FFDIE.VDHVEPRDRLLFRWSFLGVGP....ECQVGWTLTGGAEATTLTVDDSCPGR...
Cterm RFTLRDW.QLDRERALTFAVE....IPGARTVTACQVRTEPGERGRTLSVSHQG..WHRL
            200         210          220         230         240
Cterm                          TT                   TT
           β11          β12                  β13         β14        η1

                          α3
Nterm
                 120         130          139
Nterm ......PGSEVAQLKAGWLDFVGRLARYLETG
Cterm GLSDLQERTLRHRFAATWTAALSLAEECAR..
        250        260         270      277
Cterm
                       α5
```

Figure S2 Superposition of the N-terminal (magenta) and C-terminal (cyan) domains of DynU16 and the resulting sequence alignment produced by the super alogrithm in *PyMOL* (v2.4.1, Schrödinger). The structural alignment was based on backbone atom positions. After refinement, the alogrithm retained 318 matched atom pairs with an RMSD of 2.0 Å. The resulting sequence alignment aligns 107 residues with 15% identity over this range. The sequence alingment was formatted using *ESPript* 3.0 (Robert & Gouet, 2014).

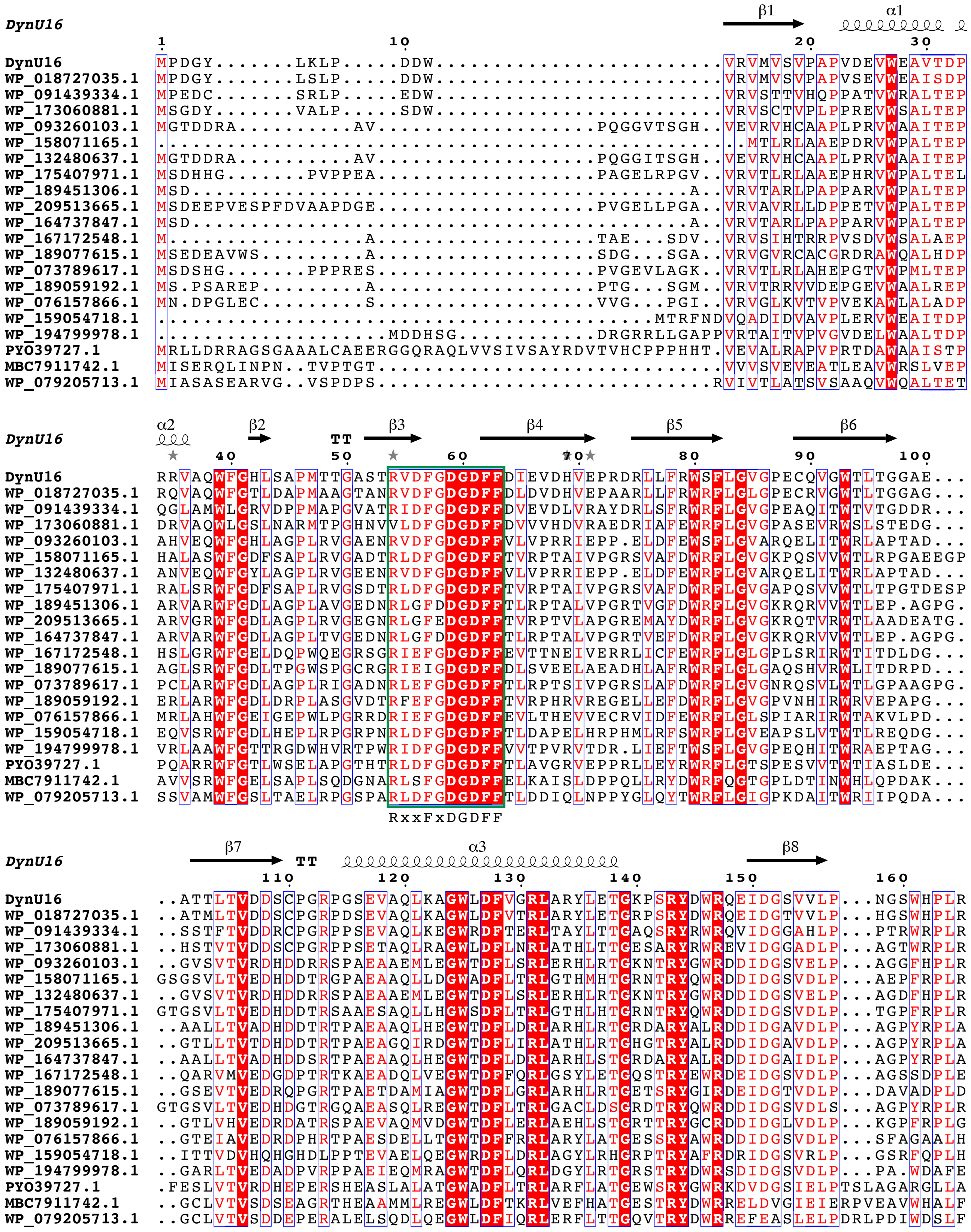

DynU16
β1 α1
1 10 20 30
DynU16 MPDGY.......LKLP.....DDW.........................VRVMVSVPAPVDEVWEAVTDP
WP_018727035.1 MPDGY.......LSLP.....DDW.........................VRVMVSVPAPVSEVWEAISDP
WP_091439334.1 MPEDC.......SRLP.....EDW.........................VRVSTTVHQPPATVWRALTEP
WP_173060881.1 MSGDY.......VALP.....SDW.........................VRVSCTVPLPPREVWSALTEP
WP_093260103.1 MGTDDRA..........AV.................PQGGVTSGH..VEVRVHCAAPLPRVWAAITEP
WP_158071165.1 ....MTLRLAAEPDRVWPALTEP
WP_132480637.1 MGTDDRA..........AV.................PQGGITSGH..VEVRVHCAAPLPRVWAAITEP
WP_175407971.1 MSDHHG.......PVPPEA.................PAGELRPGV..VRVTLRLAAEPHRVWPALTEL
WP_189451306.1 MSD..................................A..VRVTARLPAPPARVWPALTEP
WP_209513665.1 MSDEEPVESPFDVAAPDGE.................PVGELLPGA..VRVAVRLLDPPETVWPALTEP
WP_164737847.1 MSD..................................A..VRVTARLPAPPARVWPALTEP
WP_167172548.1 M.............A.....................TAE...SDV..VRVSIHTRRPVSDVWSALAEP
WP_189077615.1 MSEDEAVWS.....A.....................SDG...SGA..VRVGVRCACGRDRAWQALHDP
WP_073789617.1 MSDSHG.......PPPRES.................PVGEVLAGK..VRVTLRLAHEPGTVWPMLTEP
WP_189059192.1 MS.PSAREP........A..................PTG...SGM..VRVTRRVVDEPGEVWAALREP
WP_076157866.1 MN.DPGLEC........S..................VVG...PGI..VRVGLKVTVPVEKAWLALADP
WP_159054718.1 ....MTRFNDVQADIDVAVPLERVWEAITDP
WP_194799978.1 ....MDDHSG...........DRGRRLLGAPPVRTAITVPVGVDELWAALTDP
PYO39727.1 MRLLDRRAGSGAAALCAEERGGQRAQLVVSIVSAYRDVTVHCPPPHHT.VEVALRAPVPRTDAWAAISTP
MBC7911742.1 MISERQLINPN..TVPTGT.......................VVVSVEVEATLEAVWRSLVEP
WP_079205713.1 MIASASEARVG..VSPDPS........................RVIVTLATSVSAAQVWQALTET
DynU16
α2 β2 TT β3 β4 β5 β6
40 50 60 70 80 90 100
DynU16 RRVAQWFGHLSAPMTTGASTRVDFGDGDFFDIEVDHVEPRDRLLFRWSFLGVGPECQVGWTLTGGAE...
WP_018727035.1 RQVAQWFGTLDAPMAAGTANRVDFGDGDFFDLDVDHVEPAARLLFRWRFLGLGPECRVGWTLTGGAG...
WP_091439334.1 QGLAMWLGRVDPPMAPGVATRIDFGDGDFFDVEVDLVRAYDRLSFRWRFLGVGPEAQITWTVTGDDR...
WP_173060881.1 DRVAQWLGSLNARMTPGHNVVLDFGDGDFFDVVHDVRAEDRIAFEWRFLGVGPASEVRWSLSTEDG...
WP_093260103.1 AHVEQWFGHLAGPLRVGAENRVDFGDGDFFVLVPRRIEPP.ELDFEWSFLGVARQELITWRLAPTAD...
WP_158071165.1 HALASWFGDFSAPLRVGADTRLDFGDGDFFTVRPTAIVPGRSVAFDWRFLGVGKPQSVVWTLRPGAEEGP
WP_132480637.1 ANVEQWFGYLAGPLRVGEENRVDFGDGDFFVLVPRRIEPP.ELDFEWRFLGVARQELITWRLAPTAD...
WP_175407971.1 RALSRWFGDFSAPLRVGSDTRLDFGDGDFFTVRPTAIVPGRSVAFDWRFLGVGAPQSVVWTLTPGTDESP
WP_189451306.1 ARVARWFGDLAGPLAVGEDNRLGFDDGDFFTLRPTALVPGRTVGFDWRFLGVGKRQRVVWTLEP.AGPG.
WP_209513665.1 ARVGRWFGDLAGPLRVGEGNRLGFEDGDFFTVRPTVLAPGREMAYDWRFLGVGKRQTVRWTLAADEATG.
WP_164737847.1 ARVARWFGDLAGPLTVGEDNRLGFDDGDFFTLRPTALVPGRTVEFDWRFLGVGKRQRVVWTLEP.AGPG.
WP_167172548.1 HSLGRWFGELDQPWQEGRSGRIEFGDGDFFEVTTNEIVERRLICFEWRFLGLGPLSRIRWTITDLDG...
WP_189077615.1 AGLSRWFGDLTPGWSPGCRGRIEIGDGDFFDLSVEELAEADHLAFRWRFLGLGAQSHVRWLITDRPD...
WP_073789617.1 PCLARWFGDLAGPLRIGADNRLEFGDGDFFTLRPTSIVPGRSLAFDWRFLGVGNRQSVLWTLGPAAGPG.
WP_189059192.1 ERLARWFGDLDRPLASGVDTRFEFGDGDFFTVRPHRVREGELLEFDWRFLGVGPVNHIRWRVEPAPG...
WP_076157866.1 MRLAHWFGEIGEPWLPGRRDRIEFGDGDFFEVLTHEVVECRVIDFEWRFLGLSPIARIRWTAKVLPD...
WP_159054718.1 EQVSRWFGDLHEPLRPGRPNRLDFGDGDFFTLDAPELHRPHMLRFSWRFLGVEAPSLVTWTLREQDG...
WP_194799978.1 VRLAAWFGTTRGDWHVRTPWRIDFGDGDFFVVTPVRVTDR.LIEFTWSFLGVGPEQHITWRAEPTAG...
PYO39727.1 PQARRWFGTLWSELAPGTHTRLDFGDGDFFTLAVGRVEPPRLLEYRWRFLGTSPESVVTWTIASLDE...
MBC7911742.1 AVVSRWFGELSAPLSQDGNARLSFGDGDFFELKAISLDPPQLLRYDWRFQGTGPLDTINWHLQPDAK...
WP_079205713.1 SSVAMWFGSLTAELRPGSPARLDFGDGDFFTLDDIQLNPPYGLQYTWRFLGIGPKDAITWRIIPQDA...
RxxFxDGDFF
DynU16
β7 TT α3 β8
110 120 130 140 150 160
DynU16 ..ATTLTVDDSCPGRPGSEVAQLKAGWLDFVGRLARYLETGKPSRYDWRQEIDGSVVLP...NGSWHPLR
WP_018727035.1 ..ATMLTVDDSCPGRPDSEVAQLKAGWLDFVTRLARYLETGRPSRYDWRQEIDGTVVLP...HGSWHPLR
WP_091439334.1 ..SSTFTVDDRCPGRPPSEVAQLKEGWRDFTERLTAYLTTGAQSRYRWRQVIDGGAHLP...PTRWRPLR
WP_173060881.1 ..HSTVTVDDSCPGRPPSETAQLRAGWLDFLNRLATHLTTGESARYRWREVIDGGADLP...AGTWRPLR
WP_093260103.1 ..GVSVTVRDHDDRRSPAEAAEMLEGWTDFLSRLERHLRTGKNTRYGWRDDIDGSVELP...AGGFHPLR
WP_158071165.1 GSGSVLTVEDHDDTRGPAEAAQLLDGWADFLTRLGTHMHTGRNTRYQWRDDIDGSVDLP...AEPFRPLR
WP_132480637.1 ..GVSVTVRDHDDRRSPAEAAEMLEGWTDFLSRLERHLRTGKNTRYGWRDDIDGSVELP...AGDFHPLR
WP_175407971.1 GTGSVLTVEDHDDTRSAAESAQLLHGWSDFLTRLGTHLHTGRNTRYQWRDDIDGSVDLP...TGPFRPLR
WP_189451306.1 ..AALLTVADHDDTRTPAEAAQLHEGWTDFLDRLARHLRTGRDARYALRDDIDGAVDLP...AGPYRPLA
WP_209513665.1 ..GTLLTVTDHDDTRTPAEAGQIRDGWTDFLIRLATHLRTGHGTRYALRDDIDGAVDLP...AGPYRPLA
WP_164737847.1 ..AALLTVADHDDSRTPAEAAQLHEGWTDFLDRLARHLSTGRDARYALRDDIDGAIDLP...AGPYRPLA
WP_167172548.1 ..QARVMVEDGDPTRTKAEADQLVEGWTDFFQRLGSYLETGQSTRYEWRDEIDGSVDLP...AGSSDPLE
WP_189077615.1 ..GSEVTVEDRQPGRTPAETDAMIAGWTDFLGRLARHLRTGETSRYGIRDEIDGTVDLP...DAVADPLD
WP_073789617.1 GTGSVLTVEDHDGTRGQAEASQLREGWTDFLTRLGACLDSGRDTRYQWRDDIDGSVDLS...AGPYRPLR
WP_189059192.1 ..GTLVHVEDRDATRSPAEVAQMVDGWTDFLERLAEHLSTGRTTRYGCRDDIDGLVDLP...KGPFRPLG
WP_076157866.1 ..GTEIAVEDRDPHRTPAESDELLTGWTDFFRRLARYLATGESSRYAWRDDIDGSVDLP...SFAGAALH
WP_159054718.1 ..ITTVDVHQHGHDLPPTEVAELQEGWRDFLDRLAGYLRHGRPTRYAFRDRIDGSVRLP...GSRFQPLH
WP_194799978.1 ..GARLTVEDADPVRPPAEIEQMRAGWTDFLQRLDGYLRTGRSTRYDWRSDIDGSVDLP...PA.WDAFE
PYO39727.1 .FESLVTVRDHEPERSHEASLALATGWADFLTRLARFLATGEWSRYGWRKDVDGSIELPTSLAGARGLLA
MBC7911742.1 ..GCLVTVSDSEAGRTHEAAMMLREGWLDFTKRLVEFHATGESTRYDWRRELDVGIEIERPVEAVWHALF
WP_079205713.1 ..GCLVTVSDDEPERALELSQDLQEGWLDFIQRLERFLTTGQVTRYDWRREFEASLELPDRLPDIWDSLF

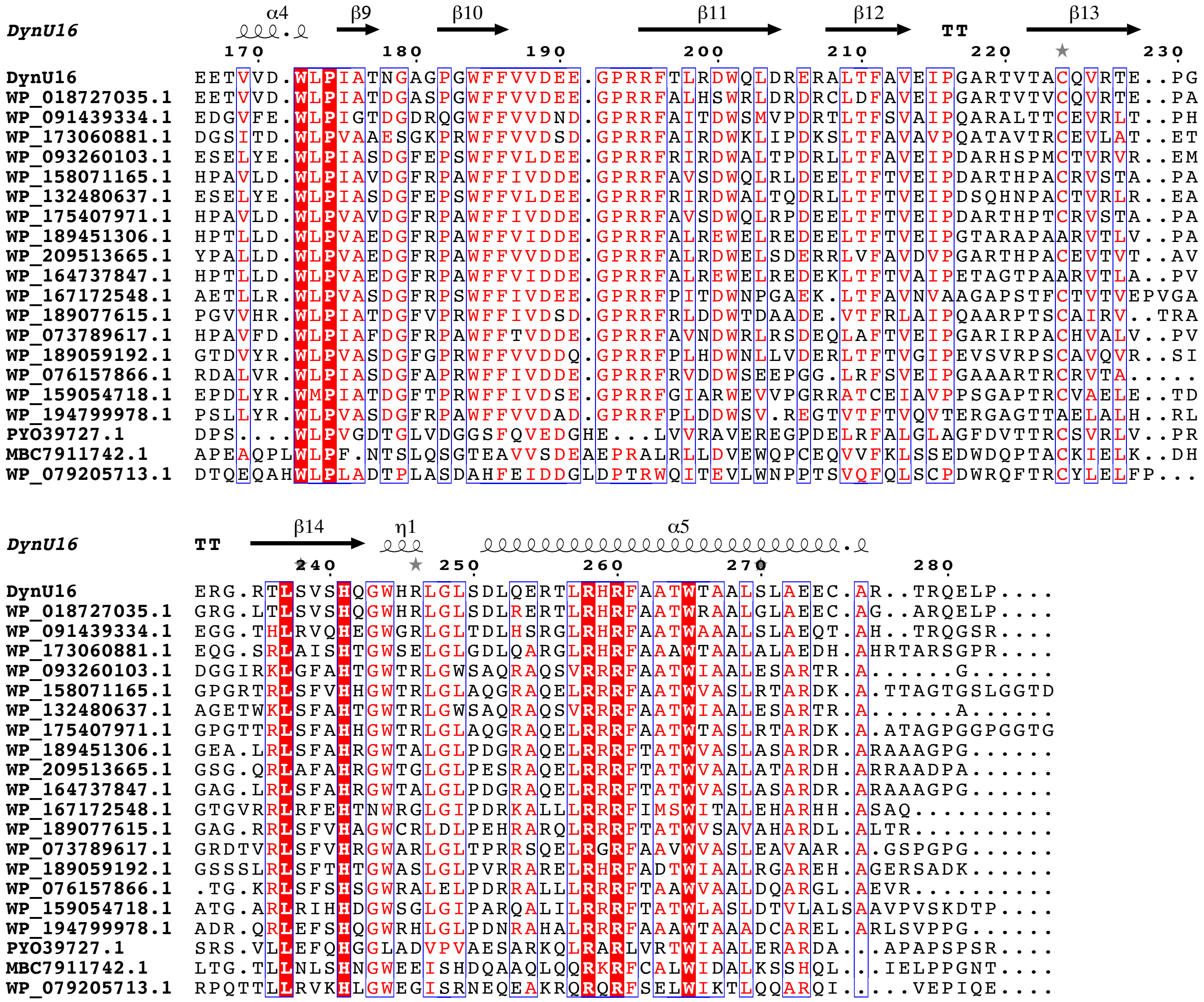

Accession	Evalue	%Id	Species
WP_018727035.1	3e-179	84%	*Salinispora pacifica*
WP_091439334.1	2e-119	60%	*Micromonospora yangpuensis*
WP_173060881.1	4e-106	56%	*Phytohabitans houttuyneae*
WP_093260103.1	1e-88	52%	*Saccharopolyspora shandongensis*
WP_158071165.1	8e-86	50%	*Streptomyces* sp. CB03234
WP_132480637.1	2e-85	50%	*Saccharopolyspora* sp. 7K502
WP_175407971.1	4e-85	50%	*Streptomyces* sp. TRM64462
WP_189451306.1	3e-80	48%	*Streptomyces abikoensis*
WP_209513665.1	1e-79	47%	*Streptomyces syringium*
WP_164737847.1	1e-79	48%	*Streptomyces luteoverticillatus*
WP_167172548.1	5e-77	45%	*Saccharomonospora amisosensis*
WP_189077615.1	2e-76	46%	*Mangrovihabitans endophyticus*
WP_073789617.1	4e-74	47%	*Streptomyces uncialis*
WP_189059192.1	2e-73	46%	*Longimycelium tulufanense*
WP_076157866.1	9e-71	45%	*Amycolatopsis coloradensis*
WP_159054718.1	2e-70	43%	*Streptomyces dysideae*
WP_194799978.1	5e-69	47%	*Micromonospora* sp. ANENR4
PYO39727.1	3e-49	36%	*Gemmatimonadetes* bacterium
MBC7911742.1	1e-48	33%	*Pyrinomonadaceae* bacterium
WP_079205713.1	1e-47	33%	*Microcystis aeruginosa*

Figure S3 Sequence alignment and blast E-values for 20 di-domain homologs to DynU16 identifed with NCBI blast (Altschul et al., 1997). These homologs have at least 90% coverage and over 30% identity and were clustered to retain only a single represenative sequence at 98% sequence idenity. The RxxFxDGDFF motif is highlighted in a green box between strands β3 and β4. Many of the residues conserved across all 21 protiens line the cavity with the majority of the residues from the N-terminal domain. Most of the remaining conserved residues stabilize packing between secondary structure elements. The alignment was formatted using *ESPript* 3.0 (Robert & Gouet, 2014).
